# Gray Wolf Exposure to Emerging Vector-Borne Diseases in Wisconsin with Comparison to Domestic Dogs and Humans

**DOI:** 10.1371/journal.pone.0165836

**Published:** 2016-11-29

**Authors:** Rocio F. Jara, Adrian P. Wydeven, Michael D. Samuel

**Affiliations:** 1 Nelson Institute for Environmental Studies, University of Wisconsin-Madison, Madison, Wisconsin, United States of America; 2 Wisconsin Department of Natural Resources, Retired, Ashland, Wisconsin, United States of America; 3 U.S. Geological Survey, Wisconsin Cooperative Wildlife Research Unit, University of Wisconsin-Madison, Madison, Wisconsin, United States of America; University of Kentucky College of Medicine, UNITED STATES

## Abstract

World-wide concern over emerging vector-borne diseases has increased in recent years for both animal and human health. In the United Sates, concern about vector-borne diseases in canines has focused on Lyme disease, anaplasmosis, ehrlichiosis, and heartworm which infect domestic and wild canids. Of these diseases, Lyme and anaplasmosis are also frequently diagnosed in humans. Gray wolves (*Canis lupus*) recolonized Wisconsin in the 1970s, and we evaluated their temporal and geographic patterns of exposure to these four vector-borne diseases in Wisconsin as the population expanded between 1985 and 2011. A high proportion of the Wisconsin wolves were exposed to the agents that cause Lyme (65.6%) and anaplasma (47.7%), and a smaller proportion to ehrlichiosis (5.7%) and infected with heartworm (9.2%). Wolf exposure to tick borne diseases was consistently higher in older animals. Wolf exposure was markedly higher than domestic dog (*Canis familiaris*) exposure for all 4 disease agents during 2001–2013. We found a cluster of wolf exposure to *Borrelia burgdorferi* in northwestern Wisconsin, which overlaps human and domestic dog clusters for the same pathogen. In addition, wolf exposure to Lyme disease in Wisconsin has increased, corresponding with the increasing human incidence of Lyme disease in a similar time period. Despite generally high prevalence of exposure none of these diseases appear to have slowed the growth of the Wisconsin wolf population.

## Introduction

World-wide concern over emerging vector-borne diseases has increased in recent years for both animal and human health [1,2]. In the United Sates (US), concern about canine vector-borne diseases is mainly focused on Lyme disease, anaplasmosis, ehrlichiosis, and heartworm [3] which infect domestic and wild canids. Infections with these pathogens can cause severe illness in domestic and wild canids, affecting survivorship of the host [4–8]. These diseases also affect humans, with Lyme disease and anaplasmosis most frequently diagnosed in the Northeast and upper Midwest US [9–12]. In Wisconsin, Lyme disease in humans has been increasing since the 1990's and nationwide Lyme disease is currently the most common vector-borne disease with more than 30,000 cases reported in 2012 [13]; however, true incidence could be much higher due to underreporting [14]. Anaplasmosis became a reportable disease in 1999, and since then the annual number of human anaplasmosis cases reported in Wisconsin has grown steadily from 1 case in 2000 to > 500 during 2010–2104 [15].

In the US, Lyme disease is primarily caused by the bacterium *B*. *burgdorferi sensu stricto* (although a novel *B*. *burgdorferi* sensu lato genospecies (candidatus *Borrelia mayonii*) has been recently described [16]) and anaplasmosis by the Rickettsiales *Anaplasma phagocytophilum* [17]. The black-legged tick *Ixodes scapularis* is the vector for both pathogens in the Midwest and Northeast [18]; therefore, coinfection may also occur [3]. White-tailed deer (*Odocoileus vriginanus*) is an important host for adult *I*. *scapularis* ticks [18] while white footed mice (*Peromyscus leucopus*) are the main reservoir for these disease agents [19–22]; however, many wild mammals, including gray wolves (*Canis lupus*), can also serve as hosts. Serological studies in Minnesota and Wisconsin found gray wolf exposure to *B*. *burgdorferi* ranging from 3% [23] to 48% [24]. *Anaplasma phagocytophilum* was reported in a captive timber wolf in Austria that showed anorexia, depression, and fever [4]. In domestic dogs (*Canis familiaris*), geographic patterns of prevalence of exposure to *B*. *burgdorferi* and *A*. *phagocytophilum* are similar to the incidence pattern in humans, i.e., higher prevalence in the upper Midwest and Northeastern US [3]. In particular, 10.2% and 10.5% of the domestic dogs tested in Wisconsin were positive for exposure to *B*. *burgdorferi* and *A*. *phagocytophilum* respectively, between 2001 and 2007 [3].

Canine ehrlichiosis is caused by the bacterium *Ehrlichia canis* [25] and transmitted primarily by the brown dog tick *Rhipicephalus sanguineus* [26]. In domestic dogs from the US, the highest prevalence has been detected in the Southeast (0.6% prevalence). In Wisconsin, where the brown dog tick is rare, the prevalence in domestic dogs is 0.3% [3]. Human infection with this pathogen has only been reported in 6 patients in Venezuela [10]. Infection with this pathogen occurred in a captive gray wolf in Florida, US, that showed epistaxis, anorexia, weight loss [7].

Heartworm is caused by the nematode *Dirofilaria immitis* transmitted by *Aedes*, *Anopheles*, and *Culex* mosquitoes. Feral dogs and coyotes (*Canis latrans*) are recognized reservoirs for this parasite [27]. Infection is widely distributed in domestic dogs in the US with the highest prevalence in the Southeast [3]. In Wisconsin, only 0.6% of domestic dogs tested positive for infection with this parasite [3]. Prevalence in wild red wolves (*Canis rufus*) in Southeastern US has reached 100%, whereas in Wisconsin, studies reported from 0% to 9% of gray wolf infection [24] (Dorothy Ginnett and Jerold Theis, unpublished report to the DNR 2003). Human infection with *D*. *immitis* can occur, and is positively correlated with the prevalence in domestic dogs at a state level [28]; however, most human cases are asymptomatic [29].

Gray wolves had become extirpated in Wisconsin in the late 1950s, but began to recolonize the state in the mid-1970s after being listed as federally endangered in 1974, and state endangered in 1975 [30]. The Wisconsin Department of Natural Resources (WDNR) began monitoring the wolf population in winter 1979–1980 when 25 wolves were counted in midwinter. Subsequently the population grew to 782 wolves by winter 2011 [30,31]. Sampling animals for disease testing was started in 1981, and archived samples of blood and serum for additional disease testing was initiated in 1985 [32]. Gray wolves were removed from the state list of endangered and threatened species in 2004 [30]. Wolves were down-listed to federally threatened in 2003, and delisted in 2007, but during recent legal battles they have returned to the federal list of endangered species [33,34]. The disease status in wild wolves is of interest in assessing the success of wolf recovery and recolonization, as well as assessing the effect of diseases on population growth and role in wolf population dynamics.

Because wolves are free-ranging and have more contact with infectious vectors, such as ticks, their probability of being exposed to these pathogens is likely higher than humans and domestic dogs. Accordingly, we expect higher wolf exposure to these pathogens than for humans and domestic dogs. Aside from a few reports in wild gray wolves, there are no large temporal and spatial scale studies of wolf exposure to these vector-borne diseases. It is useful to study vector-borne exposures in wolves to better understand health threats to wolf populations, but also because wolves can serve as a sentinel species for human and domestic dog health risk. Wild sentinels may be more likely to reflect ecological changes in disease incidence than public health reporting because diagnostic reporting and case definitions used to diagnose human Lyme disease change over time. In addition, preventive veterinary medicine (e.g., vaccination for Lyme disease and anthelmintics), diagnostic testing (annual exposure testing using SNAP 4Dx Test and similar methods), tick removal, and antibiotic treatment mean that domestic dogs may provide a biased assessment of natural pathogen exposure or risk. In addition, wolves might better indicate zones of higher risk because domestic dog movements coincide with their owners (i.e., they visit similar areas and spend similar amounts of time in tick friendly habitats). In Wisconsin, it is possible to integrate varied sources of information including tick and mouse field surveys (e.g., for Lyme disease and anaplasmosis), human case reports, and domestic and wild host prevalence reports [35].

The objective of our study is to evaluate the temporal and geographic patterns of gray wolf exposure to agents causing four vector-borne diseases—Lyme disease, anaplasmosis, canine ehrlichiosis and heartworm—in Wisconsin between 1985 and 2011. Specifically, we measured seroprevalence in gray wolves over time and identified clusters of high exposure to these diseases. In addition, we compared temporal and geographic patterns in wolves to humans and domestic dogs to evaluate similarity in patterns of exposure/infection.

## Material and Methods

We conducted this study using 373 blood or sera samples from wild Wisconsin wolves collected between 1985 and 2011. The samples were from wolves captured as part of the annual monitoring program conducted by the Wisconsin Department of Natural Resources (WDNR) [30] or captured for depredation management by the U.S. Department of Agriculture Wildlife Service (USDA-WS) [36]. Wolves captured as non-target species during the coyote trapping season or found dead from various causes (vehicle collision, illegally killed, diseases) were also included. Along with collecting sera samples, wolves were examined for ectoparasites, and both *Dermacentor* and *Ixodes* ticks were commonly found on wolves. The samples came from 45 of 72 counties, representing mainly the northern forest region of Wisconsin (primary habitat for gray wolves in the state). Blood samples of 10 cc or less were obtained from the cephalic, saphenous, femoral, or jugular veins of live (captured/released) wolves using needles of gauges 20–25 (20–22 on adults, 22–25 on pups or immature adults). Samples collected by the WDNR were obtained following a standard operating protocol (SOP) approved by the WDNR Animal Care and Use Committee (ACUC). The USDA-WS followed a separate SOP, also approved by the WDNR ACUC, for wolves captured for depredation management.

Wolves captured by the WDNR for monitoring and research purposes were mostly captured on national, state, or county forest, as well as state wildlife areas with permission of the U.S. Forest Service or other public land managers. A few wolves were captured for monitoring by the WDNR on private lands with authorization of the landowner. When federally listed as threatened or endangered WDNR capture and monitoring of wolves was done under authority of permits from the U.S. Fish and Wildlife Service. Most wolves captured at depredation site by USDA-WS, operating under the authority of the WDNR, were captured on private land with permission of the landowner. Prior to 2003, while wolves were listed as federally endangered, depredating wolves were captured by USDA-WS and translocated to the Chequamegon-Nicolet National Forest or Menominee Indian reservation by WDNR with approvals by the Forest Supervisor or Tribal Chairperson. After 2003, most depredating wolves were euthanized by USDA-WS under threatened status listing authority, special permits, or delisting authority as granted by the U.S. Fish and Wildlife Service. Euthanized depredating wolf specimens were used for scientific, educational, or cultural (tribal) uses.

We tested blood (stored at room temperature on Nobuto strips) and sera samples (stored frozen) using the SNAP 4Dx Test (IDEXX Laboratories, Westbrook, ME, USA) to determine exposure of gray wolves to the four vector-borne disease agents. This test, designed for domestic dogs, detects antibodies to *B*. *burgdorferi*, *E*. *canis*, and *A*. *phagocytophilum* indicating past or current exposure; and the antigen for *D*. *immitis*, indicating current infection. Because the test was designed for domestic dogs, we conducted a validation study for gray wolves [37] by comparing frozen wolf serum with previous diagnostic results to those from the SNAP 4Dx Test (S1 Text). We found near perfect agreement for Lyme disease (94.1% n = 17). However, the agreement was weaker for both anaplasmosis (64.7% n = 17) and heartworm (68.5% n = 35) suggesting our study might underestimate prevalence of exposure to *A*. *phagocytophilum* and infection with *D*. *immitis* in Wisconsin wolves. Unfortunately, none of the samples was previously tested for antibodies against *E*. *canis* so validation for this agent was not conducted.

### Statistical analysis

We report prevalence of wolf exposure to these four pathogens (positive wolves/number tested). We used separate logistic regression analysis to determine which predictor variables best explain the prevalence of exposure to each pathogen. The response was the presence (1) or absence (0) of antibodies or antigen (for heartworm) assessed with the SNAP 4Dx Test. The predictor variables were age (pup, yearling, and adult), sex (male and female), and year (from 1985 to 2011). For *E*. *canis*, none of the pups tested positive. Therefore, we used a Z-test and Bonferroni’s multiple comparisons to test for differences in % of exposure between pups, yearlings, and adults. For each pathogen, we considered all 2-way interactions between the predictor variables. We used backward elimination to arrive at a final model, dropping the least significant variables with P > 0.05. We used χ^2^ tests to determine goodness of fit of the final models. To test for coexposure between *B*. *burgdorferi* and *A*. *phagocytophilum*, we used Pearson’s Chi-squared test of independence with Yates’ continuity correction to evaluate potential association between exposures to both pathogens.

To identify geographic clusters of disease, we used the Bernoulli spatial scan statistics [38] implemented in SaTScanTM. We assigned each wolf to the county of capture and performed spatial analysis for each disease at this geographical scale. We conducted the spatial analysis for gray wolves, domestic dogs, and humans. We used the discrete Poisson model to analyze domestic dogs using the cases for each pathogen and population at risk in every county which we estimated using the formula provided by The American Veterinary Association [39]. For humans we also used the discrete Poisson model to identify clusters of Lyme disease cases, the only pathogen for which we had human data. We used logistic regression to further analyze temporal trends in wolf exposure within the cluster and linear regression to analyze the annual trend in human incidence within clusters. The response variable for the linear regression was human incidence (100,000× cases /population) of Lyme disease and year (as a continuous variable) was the explanatory variable.

To compare domestic dogs to gray wolves we used two sources of domestic dog data: prevalence of exposure and number of cases. Bowman et al. (2009) [3] reported the prevalence of exposure to *B*. *burgdorferi*, *A*. *phagocytophilum* and *E*. *canis* or infection with *D*. *immitis* for dogs in Wisconsin between 2001 and 2007 (no heartworm results for 2002 and 2003). The SNAP 4Dx Test in that study is the same that we used for testing wolves. We used a Z-test with normal approximation to compare proportions of positive results between wolves and domestic dogs at a state level. We only used wolf data between 2001 and 2007 to make it comparable to the domestic dog data set. The IDEXX laboratory website [40] provided positive domestic dog cases by county between 2007 and 2013 (the county identifies where the individual was tested). We used this data to compare spatial clustering between wolves and domestic dogs. For the comparison of disease patterns in gray wolves and humans, we used the incidence (cases/total population) of Lyme disease for humans in Wisconsin counties between 1989 and 2011 (Personal communication, Kristin Hardy, Wisconsin Department of Health Services).

## Results

### Wolf exposure to vector-borne disease agents

**Fig 1:**
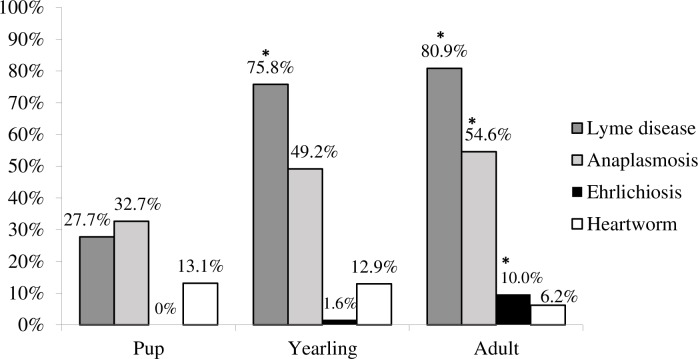
Wolf seroprevalence to *B*. *burgdorferi*, *A*. *phagocytophilum*, *E*. *canis*, and *D*. *immitis* by age group in Wisconsin during 1985 to 2011. Pup represents individuals between 0 and 1 year old, yearling between 1 and 2 years old and adult are individuals older than 2 years old. Asterisks represent significant differences (P<0.05) compared to pups. Sample size for pup = 99–101, yearling = 62–63, adult = 207–210 depending on the disease.

**Fig 2:**
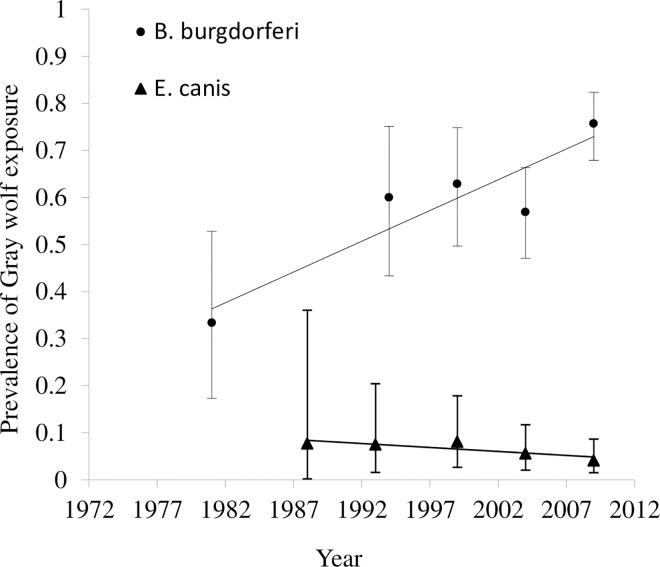
Temporal trend in wolf seroprevalence to *B*. *burgdorferi* and *E*. *canis* in Wisconsin during 1985 to 2011. For both pathogens years were grouped into the following 5 groups: 1985–1990 (n = 13), 1991–1996 (n = 40), 1997–2001 (n = 62), 2002–2006 (n = 109 and 108 for *B*. *burgdorferi* and *E*. *canis*, respectively), and 2007–2011 (n = 148 and 147 for *B*. *burgdorferi* and *E*. *canis*, respectively). Data points represent the observed prevalence of wolf exposure within a period. Vertical bars represent 95% confidence interval (CI). Coefficient estimate for *B*. *burgdorferi* = -0.05 (P = 0.03, 95% CI = 0.0–0.09, Odds Ratio = 1.05, 95% CI = 1.0–1.05). Coefficient estimate for *E*. *canis* = -0.09 (P = 0.026; CI = -0.16 –-0.01, Odds Ratio = 0.92, CI = 0.85–0.99).

We found prevalence of exposure to vector-borne disease agents in Wisconsin wolves between 1985 and 2011 was 65.6% (n = 372) for *B*. *burgdorferi*, 47.7% (n = 369) for anaplasma, 5.7% (n = 369) for ehrlichia, and 9.2% (n = 371) for heartworm. There was no difference in exposure between males and females for any of the diseases (Tables 1 and 2). Logistic regression for *B*. *burgdorferi* showed that age and year were significant predictors of wolf exposure (Table 1). The proportions of adults (80.9%, Odds Ratio (OR) = 10.8, 95% Confidence interval (CI) = 6.24–19.17) and yearlings (75.8%, OR = 9.41, CI = 4.51–20.69) exposed to *B*. *burgdorferi* were markedly higher than pups (27.7%). However adult and yearling exposure was similar (OR = 1.19, CI = 0.57–2.38) (Fig 1). We found evidence of increasing *B*. *burgdorferi* seroprevalence between 1985 and 2011 (coefficient estimate = 0.05; z = 2.17; P = 0.03; OR = 1.05, CI = 1.0–1.1) (Fig 2) indicating a 50% increase in the proportion of wolves exposed to this pathogen per decade. The model provided a good fit for the data (χ^2^ = 11.2, P = 0.27).

**Table 1:** Parameter estimates for logistic regression models of seroprevalence to *Borrelia burgdorferi*, *Anaplasma phagocytophilum*, and *Dirofilaria immitis*.

	Pup (β_0_)	Yearling (β_1_)	Adult (β_2_)	Year (β_3_)	Sex (β_4_)
*B*. *burgdorferi*	-99.62^*^ (-189.43–-10.26)	2.24^***^ (1.51–3.03)	2.38^***^ (1.83–2.95)	0.05^*^ (0–0.09)	0.44^**†**^ (-0.05–0.95)
*A*. *phagocytophilum*	-0.71^***^ (-1.14–-0.3)	0.68† (0.001–1.34)	0.89^***^ (0.4–1.4)	0.01 (-0.02–0.05)	0.32 (-0.09–0.74)
*D*. *immitis*	-2.34^***^ (-2.71–-2.0)	0.42 (-1.03–1.81)	0.85^**†**^ (-0.07–1.97)	0.06 (-0.02–0.14)	0.22 (-0.5–0.95)

Symbols represent P values

† = 0.05<P<0.1

* = 0.01<P value<0.05

*** = P value<0.001. 95% confidence intervals for each parameter are given in parenthesis. Significant coefficients estimates (P<0.05) derived from the final model with the non-significant variables removed.

**Table 2:** Parameter estimates for logistic regression models of seroprevalence to *Ehrlichia canis*.

	Intercept (β_0_)	Age (β_1_)	Year (β_2_)	Gender (β_4_)
*E*. *canis*	161.38^*^ (6.62–310.29)	2.53^**^ (1.11–5.38)	-0.09^*^ (-0.16–-0.01)	0.65 (-0.27–1.60)

Asterisks represent P values.

* = 0.01<P value<0.05

** = 0.001<P value<0.01. 95% confidence intervals for each parameter are given in parenthesis. Significant coefficients estimates (P<0.05) derived from the final model with the non-significant variables removed.

For *A*. *phagocytophilum*, age was the only significant predictor of prevalence (Table 1). The proportions of adults (54.6%; OR = 2.44, CI = 1.49–4.05) exposed to *A*. *phagocytophilum* were significantly higher than pups (32.7%) (Fig 1). Exposure to this pathogen in Wisconsin has not significantly changed over time (Coefficient estimate = 0.01, z = 0.72, P = 0.473, OR = 1.01, CI = 0.98–1.05). The model for *A*. *phagocytophilum* was a good fit to the data (χ^2^ = 7.29, P = 0.607).

The seroprevalence of *E*. *canis* was significantly associated with age and year (Table 2). Older animals are more likely to have been exposed to this pathogen (coefficient estimate = 2.53; z = -2.22, P = 0.026; OR = 13.78, CI = 3.23–242.22). Specifically, adult exposure (9.7%) was significantly higher than pup exposure (0%) (z = 3.28, Bonferroni P = 0.004); but similar to yearling exposure (1.6%, z = 2.14, Bonferroni P = 0.11) and yearling exposure (1.6%) was similar to pup exposure (z = 1.3, Bonferroni P = 0.6) (Fig 1). Since 1985, the proportion of wolves exposed to *E*. *canis* within Wisconsin has decreased 80% per decade (coefficient estimate = -0.09; z = -2.22; P = 0.026; OR = 0.92, CI = 0.85–0.99) (Fig 2). The model was a good fit for the data (χ^2^ = 9.47, P = 0.395).

Finally, none of the predictor variables or 2-way interactions was associated with heartworm infection (Table 1). These findings indicate no difference in exposure between pups (13.1%), yearlings (12.9%), and adults (6.2%) (Fig 1); or between males (8.4%) and females (8.8%). In addition, the temporal trend was not significant (Table 1) indicating that wolf infection with *D*. *immitis* did not changed significantly between 1985 and 2011. The model with only an intercept was also a good fit for the data (χ^2^ = 3.17, P = 0.957).

We found a positive association between exposure to *B*. *burgdorferi* and *A*. *phagocytophilum* (χ^2^ = 42.553, df = 1, P < 0.0001). We found that individuals that have been exposed to one pathogen have higher chances of have being exposed to the second pathogen (OR = 4.73); 39% of wolves have been exposed to both pathogens (either subsequently or simultaneously) and 26.3% were negative for both pathogens, compared to 26% exposure to *B*. *burgdorferi* alone and 8.4% to *A*. *phagocytophilum* alone.

**Fig 3:**
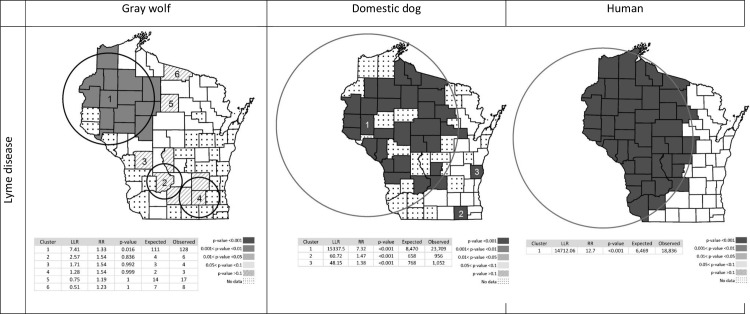
Spatial distribution of clusters of wolf seroprevalence to *B*. *burgdorferi* (1985–2011) and domestic dogs (2007–2013), and human cases (1989–2011) in Wisconsin. The maps show the location and extent of the most likely cluster and secondary clusters of seroprevalence and the counties encompassed by it are shaded. In addition, the log likelihood ratio (LLR), relative risk (RR), significance (P-value), expected number of cases (Expected) and observed number of cases (Observed) are shown for each cluster.

**Fig 4:**
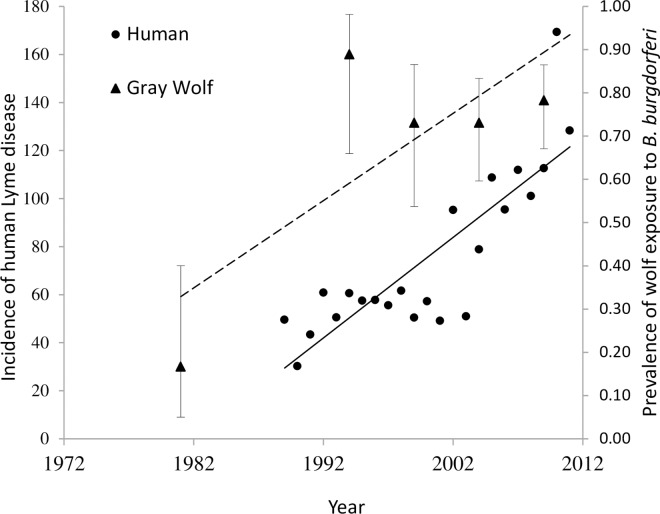
Seroprevalence of gray wolves to *B*. *burgdorferi* (Lyme disease) during 1985 to 2011, and incidence of human Lyme disease from 1989 through 2011, within spatial cluster of exposure located in northwestern Wisconsin. Wolf samples are grouped into 5-year groups (1985–1990 (n = 4), 1991–1996 (n = 18), 1997–2001 (n = 26), 2002–2006 (n = 52) and 2007–2011 (n = 69)). Data points represent the observed prevalence of wolf exposure within a period. Vertical bars represent 95% confidence interval (CI). Coefficient estimate = 0.07 (z = 1.71, P = 0.09, 95% CI = -0.01–0.15, OR = 1.07, CI = 0.99–1.17). For human Lyme disease incidence the coefficient estimate = 4.18 (t = 7.067, P< 0.001, 95% CI = 2.95–5.42).

Cluster analysis of wolf exposure to *B*. *burgdorferi* showed one significant cluster located in northwestern Wisconsin (P = 0.016), centered in Barron County with a radius of 124.3 km (Fig 3). There were 9 counties contained in the cluster: Barron, Polk, Washburn, Dunn, Chippewa, Burnett, Sawyer, Eau Claire, Pepin, Taylor, Rusk, Douglas, Price and Clark. The prevalence of wolf exposure to *B*. *burgdorferi* within and outside the cluster was 76% and 57%, respectively. Within this geographic cluster, even though not statistically significant, the proportion of wolves exposed to *B*. *burgdorferi* shows an increasing trend of 70% per decade (Coefficient estimate = 0.07, z = 1.71, P = 0.09, OR = 1.07, CI = 0.99–1.17) (Fig 4). In addition, adults (90.5%) and yearlings (85.2%) within the cluster had significantly higher exposure than pups (40.4%); however, yearlings and adults were similar (pups vs. yearling OR = 15.4, CI = 4.18–73; pups vs. adults OR = 16.5, CI = 6.66–45.15; yearlings vs. adults OR = 1.03, CI = 0.23–3.9). There was no difference in exposure within the cluster between males (74.7%) and females (78.6%) (OR = 1.6, CI = 0.69–3.9).

In contrast, there was no temporal change outside the cluster in northern Wisconsin (primary region of wolf distribution within the state), although the prevalence of exposure has varied over time (5 time periods) around an overall prevalence of 54.9% (Coefficient estimate = 0.03, z = 1.22, P = 0.221, OR = 1.03, CI = 0.99–1.07). Outside the cluster, older wolves were also more likely to be exposed to Lyme disease than pups (pups vs. yearling OR = 12.7, CI = 3.79–50.03; pups vs. adults OR = 18.59, CI = 6.69–62.13; yearlings vs. adults OR = 1.44, CI = 0.49–4.04). There was no difference between males (49.4%) and females (64.5%) (OR = 2.12, CI = 0.95–4.94).

Exposure to *A*. *phagocytophilum* and *E*. *canis* and infection with *D*. *immitis* seems to be homogeneous within the state we found no significant clusters of these agents across Wisconsin (S1 Fig). These patterns indicate that risk of exposure is equally likely throughout the state.

### Comparison of Domestic Dog and Wolf serology

In Wisconsin domestic dog exposure (number positive/number tested) between 2001 and 2007 [3] follows a similar pattern to that of wolves; higher exposure to *B*. *burgdorferi* (10.2%) and *A*. *phagocytophilum* (10.5%), but low exposure to *E*. *canis* (0.3%) and infection with *D*. *immitis* (0.6%). However, wolf exposure during the same period was markedly higher than domestic dog exposure for *B*. *burgdorferi* (61%: z = 22.18, P < 0.0001), *A*. *phagocytophilum* (42.3%: z = 13.63, P < 0.0001), *E*. *canis* (6.9%: z = 14.65, P < 0.0001), and heartworm (10.7%: z = 17.84, P < 0.0001) (S2 Fig).

There were three highly significant clusters of domestic dog exposure to *B*. *burgdorferi* in Wisconsin. The most important cluster was centered in Dunn County with a radius of 262.5 km including 29 counties of western Wisconsin (Fig 3). This cluster of Lyme disease in domestic dogs encompasses the wolf cluster. Two secondary Lyme clusters for domestic dogs included only one county each and were located in eastern and southeastern Wisconsin (Fig 3). Spatial clusters of exposure to the other three pathogens are shown in supporting information (S1 Fig).

### Human Clusters of Lyme Disease

The spatial analysis of human Lyme disease in Wisconsin revealed one significant cluster of exposure (Fig 3). The cluster is centered in Pepin County with a radius of 255.4 km and includes more than half of the counties in the state, encompassing both wolf and domestic dog clusters of Lyme disease exposure. Linear regression showed that human Lyme disease within the cluster has been increasing 42 cases per decade between 1989 and 2011 (coefficient estimate = 4.1849, t = 7.067, P < 0.001) (Fig 4).

## Discussion

A high proportion of the Wisconsin wolves were exposed to *B*. *burgdorferi* (65.6%) and *A*. *phagocytophilum* (47.7%), and a smaller proportion to *E*. *canis* (5.7%) and infected with *D*. *immitis* (9.2%) between 1985 and 2011. Exposure to *B*. *burgdorferi* between 1988 and 1996 (57.4%) and infection with *D*. *immitis* between 1991 and 1996 (2.5%) correspond with a previous study based on a subset of the Wisconsin wolves for those time periods (47.8% and 2% for *B*. *burgdorferi* and *D*. *immitis* respectively) [24]. Adult infection with *D*. *immitis* in 2003 (25%) and pup infection in 2001 (15.4%) are also similar to a previous report (9%) in Wisconsin (D. Ginnett and J. Theis, unpublished WDNR 2003 report), although there may be overlap in samples between both studies. We also found strong evidence of a positive association of gray wolf coexposure to *B*. *burgdorferi* and *A*. *phagocytophilum*. These pathogens share vector (ticks) and reservoir hosts (rodents); thus, this association is not surprising. The potential impact of coinfection/coexposure on wolves has not been studied; however, because *A*. *phagocytophilum* maybe an immunosuppressive agent, its presence could alter the intensity and duration of *B*. *burgdorferi* infection [41–43].

Wolf seroprevalence is higher in adults than pups for *B*. *burgdorferi*, *A*. *phagocytophilum* and *E*. *canis*. There are two likely explanations for these patterns. First, if antibodies to these agents are long lived, then adults have a longer risk of exposure than much younger pups. Second, questing ticks transmitting these pathogens are sedentary; therefore, exposure to tick-borne pathogens likely increases with mobility of the host. Because pups stay near den sites while adults move greater distances tick-borne diseases are expected to be higher in older animals which are more likely to contact questing ticks. The fact that yearling exposure is similar to adults suggests early exposure in life is common and movement may be an important risk factor. In contrast, *D*. *immitis*, which has similar exposure rates for all ages, is transmitted by mosquitoes, which actively search for a host.

We also found wolf exposure to *B*. *burgdorferi* has increased 50% per decade, and *E*. *canis* has decreased 80% per decade, while *A*. *phagocytophilum* exposure and *D*. *immitis* infection have remained steady since 1985. Increased wolf exposure to *B*. *burgdorferi* might be due to geographic expansion of endemic areas of infection, changes in the distribution of *I*. *scapularis*, and/or increased nymph density [44,45]. A cluster of Lyme disease is located in the area where *I*. *scapularis* was first established in Wisconsin in 1968 [46], deer density is higher (compared to other zones within wolf range) [47], and the habitat is suitable for *I*. *scapularis* [35]. Consequently, *I*. *scapularis* is abundant in this area compared to other portions of the state, Lyme disease has increased since it was first reported in the late 1960s in northwestern Wisconsin, tick density has also increased, and tick populations have expanded southward and eastward [45,46,48]. Higher tick density and expanded geographic distribution generally agrees with the higher proportion of wolves exposed to *B*. *burgdorferi* over time.

There is no clear explanation why adult wolves have a relatively high and yet declining exposure to *E*. *canis* compared to dogs when the vector (*R*. *sanguineus*) is limited by cold winter temperatures, typically found in domestic environments, and the primary reservoir (domestic dogs) of this pathogen is widely distributed in Wisconsin. One potential explanation is the discovery of the *E*. *muris*-like agent in humans and ticks in Wisconsin. This new agent was first discovered in humans in 2009 [49] with subsequent human infections reported between 2007 and 2013 [50]. A retrospective study of *Ixodes* ticks from northwestern Wisconsin showed a 1% infection rate as early as the mid-1990s [51]. In 2011 a dog in Minnesota was diagnosed with an *E*. *muris*-like agent infection, indicating this agent is present in the upper Midwest and can be pathogenic to canines [52]. These observations suggest that Wisconsin wolves may be exposed to ticks infected with the *E*. *muris*-like agent, potentially producing cross-reacting antibodies on the SNAP 4Dx *E*. *canis* test. However, SNAP 4Dx test has not been evaluated for *E*. *muris*-like agents and the infected dog in Minnesota was only positive for *Anaplasma* [52]. Further research is needed to understand the apparent decline in *E*. *canis* exposure in wolves, whether and when wolves have been exposed to *E*. *muris*, and whether *E*. *muris* produces clinical infection in wild wolves.

We also note the potential for antibody sensitivity to decline with longer storage of both frozen serum and Nobuto strips [53]. We were unable to formally assess potential degradation in our study; however, as part of the SNAP test validation experiment (S1 Text), we obtained similar results for *B*. *burgdorferi* serology from samples that were retested after up to 9 years in storage. Nevertheless, we acknowledge that our results may underestimate increasing trends in seroprevalence to these agents in the event of antibody degradation over time.

Stable exposure of wolves to *A*. *phagocytophilum* may be partly attributed to the potential for *B*. *burgdorferi* to mask exposure to *A*. *phagocytophilum* by reducing antibody titers; a pattern demonstrated in co-infected mice [54]. This masking effect may have reduced detection of *A*. *phagocytophilum*, especially considering that wolf exposure to *B*. *burgdorferi* in Wisconsin is very high, has increased, and we found coexposure between these two pathogens.

According to the SNAP 4Dx manufacturer [55] the analyte that detects exposure to *A*. *phagocytophilum* can cross react with *Anaplasma platys*, which infects domestic dogs, but its vector has not been conclusively determined [56]. In the US, infection with *A*. *platys* and *E*. *canis* are typically similar in domestic dogs, with higher prevalence in southern areas of the country [57]. Because wolf exposure to *E*. *canis* in Wisconsin is much lower (5.7%) than *A*. *phagocytophilum* (47.6%), we suspect only a small portion of the positive *A*. *phagocytophilum* results can be attributed to *A*. *platys* exposure.

Heartworm infection also remained unchanged during our study. Several factors can affect prevalence of heartworm including temperature [27]. Accordingly, infection with this parasite is more common in southern areas where higher temperatures favor mosquito transmission. These patterns suggest that further research is need to determine if higher temperatures due to climate change might increase future heartworm prevalence in domestic and wild canids in Wisconsin.

### Wolves, Domestic Dogs, and Humans

Wolf exposure to *B*. *burgdorferi*, *A*. *phagocytophilum*, *E*. *canis* and infection with *D*. *immitis* between 2007 and 2011 was significantly higher than domestic dogs to these pathogens. This difference likely arises because domestic dogs spend less time outdoors (less exposure time) and because owners use preventive methods to remove ticks and/or have domestic dogs vaccinated to reduce pathogen transmission. We believe that captured wolves represent an unbiased sample of animals for natural exposure to these diseases. Wolves captured for monitoring and research purposes were generally captured in heavily forested portions of the state where most wolves live [58]. While wolves living in mixed forest/farmland areas were less likely to be sampled, these wolves also represent a small percentage of the wolf population, that were more likely captured at livestock depredation sites [36]. We believe these samples provide a representative sample of the distribution of Wisconsin wolves.

There is high domestic dog and wolf exposure, and human infection with *B*. *burgdorferi* and *A*. *phagocytophilum* in Wisconsin, which is considered an endemic area for these diseases [3,13,59]. In addition, geographic patterns of exposure are similar between gray wolves, domestic dogs, and humans for *B*. *burgdorferi* and *A*. *phagocytophilum*. During our study period, wolf exposure to *A*. *phagocytophilum* increased only slightly and non-significantly. This finding is not consistent with the increasing number of human anaplasmosis cases reported each year in the US since the mid-1990s [59]. Increasing human anaplasmosis might be due to increased recognition of this disease over time or changes in human behavior that increase risk of exposure (e.g. spending more time in tick friendly habitat).

The increasing temporal trend of wolf exposure to *B*. *burgdorferi* corresponded with human incidence of Lyme disease, although the rise in human incidence was primarily after 2001[60], perhaps part due to under-reporting of human Lyme disease in the 1990s, followed by an exponential “catch-up” phase after 2001 as increased reporting began to reflect the true (higher) incidence. Ultimately, the increase in human cases is likely driven by ecological changes in disease/vector density and transmission. Within the overlapping geographic cluster of Lyme disease for wolves, domestic dogs, and humans in northwest Wisconsin, human incidence has increased and wolf prevalence shows an increasing trend over time. This suggests the increasing trend in human incidence in northern Wisconsin could be a consequence of ecological changes in the vector or mammalian hosts and not simply increased recognition by physicians. Domestic dog and human clusters are twice as big as the wolf cluster; however, all three clusters are centered approximately in the same areas (Barron, Dunn, and Pepin counties). The differences in cluster size between wolves, domestic dogs, and humans might in part be due to the limited range of wolves in Wisconsin compared to humans and domestic dogs. In addition, domestic dog and human records of disease are based on the county of residence and might not represent the exact location were the infection was acquired.

The geographic pattern of exposure to *E*. *canis* is similar between gray wolves and domestic dogs (S1 Fig). We found partial overlap between wolf and domestic dog clusters in northwestern Wisconsin; however, the wolf cluster is not significant. In Wisconsin there is generally low wolf and domestic dog exposure to this pathogen compared to domestic dogs from southern US [3]. It is surprising that wolf exposure to this pathogen was much higher than domestic dogs between 2001 and 2007 because the brown dog tick, which transmits *E*. *canis*, is adapted to life indoors [61]. Therefore, we expected domestic dogs to have higher exposure to this pathogen; however, tick removal or preventive treatment by owners might reduce pathogen transmission. The SNAP 4Dx Test used to detect *E*. *canis* antibodies can also cross react with *Ehrlichia chaffeensis* and *Ehrlichia ewingii* [55,62]. However, exposure to these two pathogens seems unlikely because they are transmitted by the Lone Star tick (*Amblyomma americanum*) [63,64] which is not currently found within wolf range, although there are scattered reports in southern Wisconsin (S. Paskewitz, personal communication) [65]. Human infection with *E*. *chaffeensis* has been reported in Wisconsin; however, many of these patients could have contracted the pathogen when traveling to higher risk areas in southern US [66]. Alternatively, wolf exposure to *E*. *canis* may actually represent exposure to *E*. *muris* which has been in Wisconsin since at least the mid-1990s [51]. More research is needed to determine whether the SNAP 4Dx test has cross-reactivity with *E*. *muris*-like agents and whether wolves have been historically exposed to *E*. *muris*.

Although heartworm infection is common in red wolves and domestic dogs from the southern US [3] it is uncommon in wolves and domestic dogs in Wisconsin, probably due to temperature, the lack of wolves in southern Wisconsin, and other constraints. For domestic dogs, significant clusters of exposure were spread throughout the state, and similarly, the non-significant wolf clusters of exposure were spread throughout wolf range in Wisconsin with almost no overlap between species (S1 Fig). While heartworm is known to occasionally cause mortality in wolves, the impact of the other vector-borne diseases in wolves is poorly known [67]. The earliest detection of Lyme in Minnesota wolves was for 1980, shortly after wolves had started entering Wisconsin from Minnesota [68]. The initial colonization of wolves into Wisconsin was along the border with Minnesota where an endemic of Lyme disease existed and high numbers of *I*. *scapularis* occur [30,68]. The wolf population in Wisconsin grew slowly initially, but by mid-1990s began displaying rapid growth, despite high prevalence of Lyme and anaplasma exposure [68]. The slow population growth during the 1980s has been attributed to outbreak of canine parvovirus and high human caused mortality [30], but perhaps some of these vector-borne diseases were also contributing factors. But despite the continued presence and expansion of Lyme and anaplasma exposure, the wolf population grew rapidly through the 1990s and early 2000s and by 2011 had grown to minimum winter count of 782 wolves [30,33], Thus, there is little evidence that these diseases are currently retarding growth of the Wisconsin wolf population.

## Conclusions

Lyme disease, anaplasmosis, ehrlichiosis, and heartworm are emerging vector-borne diseases that affect canids, but their prevalence in wild gray wolves has not been systematically assessed. We show Wisconsin wolves have high rates of exposure to *B*. *burgdorferi* and *A*. *phagocytophilum*, the agents causing Lyme disease and anaplasmosis, respectively. In contrast, wolves have limited exposure to *E*. *canis* and *D*. *immitis*, the agents causing canine ehrlichiosis and heartworm, respectively. These patterns are similar to domestic dogs even though wolves are constantly exposed to the vectors transmitting these pathogens. Wolf exposure to *B*. *burgdorferi* in Wisconsin has increased, corresponding with the increasing human Lyme incidence, and suggesting there might have been underreporting of the disease in humans before 2001. In addition, there is a cluster of wolf exposure to *B*. *burgdorferi* in northwestern Wisconsin, which overlaps human and domestic dog clusters for the same pathogen. The high prevalence of *B*. *burgdorferi* and *A*. *phagocytophilum* exposure, and probable underestimation of the latter and heartworm infection, suggest that these diseases could represent a possible health risk to wolf populations, although information on the effects of these diseases on wolves is lacking. During our study period the estimated winter wolf population increased from 14–16 in 1985 to 782–805 in 2011 as occupied wolf range increased >20 fold [30,31], suggesting that vector-borne disease agents have not limited population growth. Due to climate change, future heartworm risk to wolves and domestic dogs may increase, new diseases may arise, and virulence of existing diseases may change in Wisconsin. Thus periodic assessment of prevalence and impact of vector-borne diseases will be an important part of future wolf conservation programs.

## Supporting Information

S1 Data Wolf Serology Data. Data elements: age, sex, County of collection, date of collection, year of collection, and SNAP 4Dx test for Lyme, anaplasmosis, ehrlichiosis, and heart worm (0 = negative and 1 = positive).
(XLSX)

S1 Fig Spatial distribution of clusters of exposure to *A*. *phagocytophilum*, *E*. *canis* and infection with *D*. *immitis* in Gray wolves (1985–2011) and domestic dogs (2007–2013) in Wisconsin. The maps show the location and extent of the most likely cluster and secondary clusters of infection and the counties encompassed by it are shaded. In addition, the log likelihood ratio (LLR), relative risk (RR), significance (P-value), expected number of cases (Expected) and observed number of cases (Observed) are shown for each cluster.
(PDF)

S2 Fig Gray wolf and dog percent positive test results (number positive/number tested) for antibodies to *Borrelia burgdorferi*, *Anaplasma phagocytophilum* and *Echrlichia canis* and antigen of *Dirofilaria immitis* in Wisconsin between 2001 and 2007. Wolf exposure to each pathogen were siginificantly greater than dog exposure at α = 0.05 level. Sample size for wolves = 178–178 and dogs = 51512–109745 depending on the pathogen.
(PDF)

S1 Text SNAP 4Dx Validation. (PDF)
