# Supplementary material for: Gray Wolf Exposure to Emerging Vector-Borne Diseases in Wisconsin with Comparison to Domestic Dogs and Humans
Source: PLoS One. 2016 Nov 29;11(11):e0165836. doi: 10.1371/journal.pone.0165836 (PMC5127498; doi:10.1371/journal.pone.0165836)
Supplement: S1 Fig — The maps show the location and extent of the most likely cluster and secondary clusters of infection and the counties encompassed by it are shaded. In addition, the log likelihood ratio (LLR), relative risk (RR), significance (P-value), expected number of cases (Expected) and observed number of cases (Observed) are shown for each cluster. (PDF) [file pone.0165836.s002.pdf]

S1 Fig. Spatial distribution of clusters of exposure to *A. phagocytophilum, E. canis* and infection with *D. immitis* in Gray wolves (1985-2011) and domestic dogs (2007-2013) in Wisconsin. The maps show the location and extent of the most likely cluster and secondary clusters and the counties encompassed by it are shaded. In addition, the log likelihood ratio (LLR), relative risk (RR), significance (P-value), expected number of cases (Expected) and observed number of cases (Observed) are shown for each cluster.

Gray wolf

Anaplasmosis

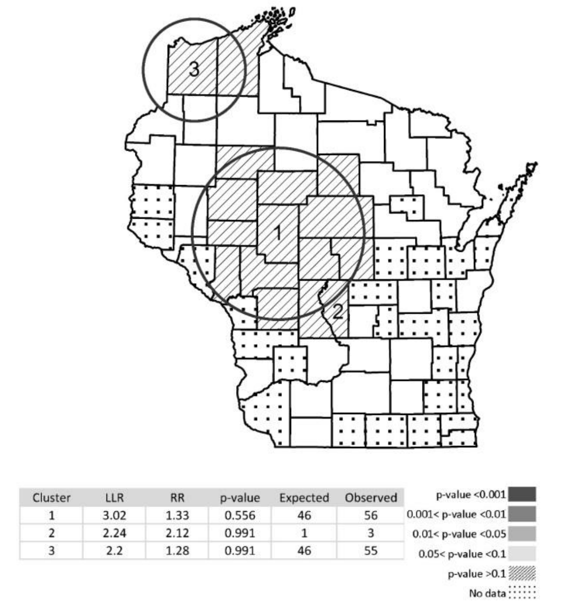

Cluster	LLR	RR	p-value	Expected	Observed
1	3.02	1.33	0.556	46	56
2	2.24	2.12	0.991	1	3
3	2.2	1.28	0.991	46	55

Ehrlichiosis

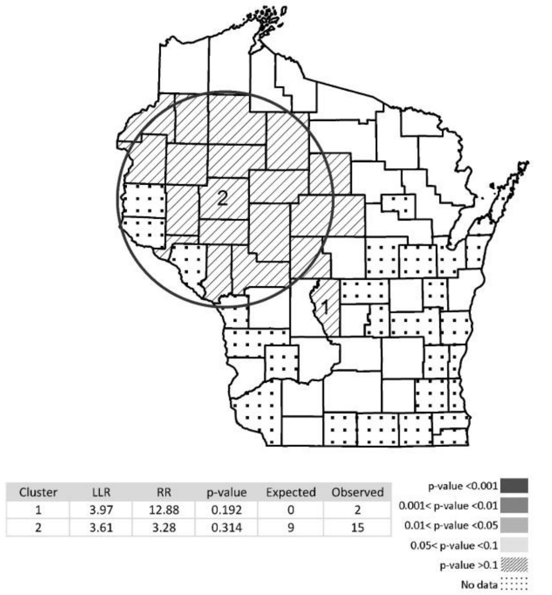

Cluster	LLR	RR	p-value	Expected	Observed
1	3.97	12.88	0.192	0	2
2	3.61	3.28	0.314	9	15

Heartworm

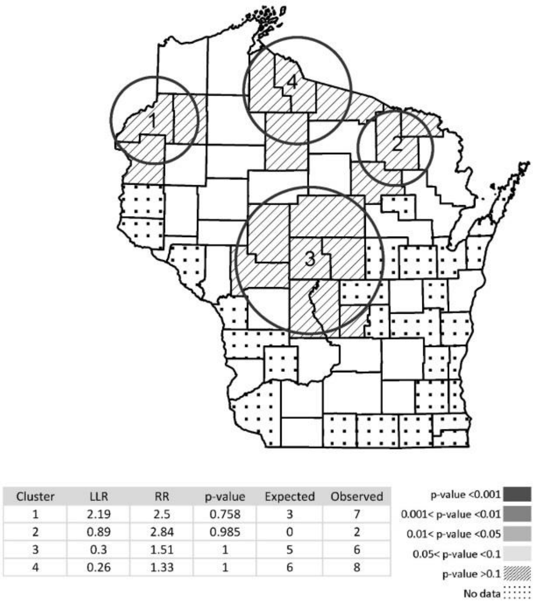

Cluster	LLR	RR	p-value	Expected	Observed
1	2.19	2.5	0.758	3	7
2	0.89	2.84	0.985	0	2
3	0.3	1.51	1	5	6
4	0.26	1.33	1	6	8

Domestic dog

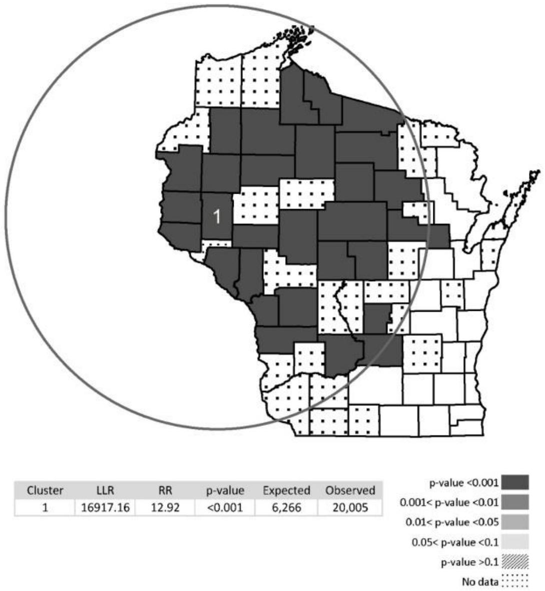

Cluster	LLR	RR	p-value	Expected	Observed
1	16917.16	12.92	<0.001	6,266	20,005

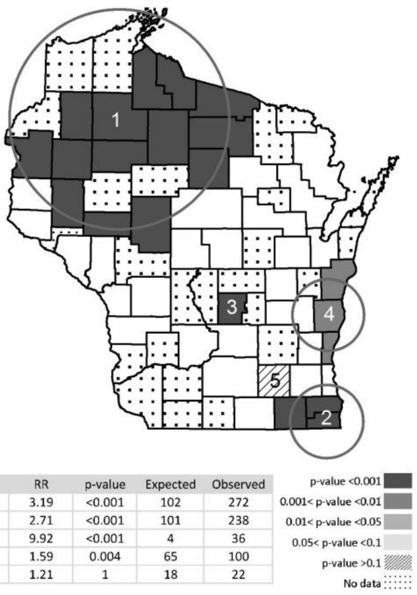

Cluster	LLR	RR	p-value	Expected	Observed
1	111.51	3.19	<0.001	102	272
2	76.25	2.71	<0.001	101	238
3	49.77	9.92	<0.001	4	36
4	8.57	1.59	0.004	65	100
5	0.37	1.21	1	18	22

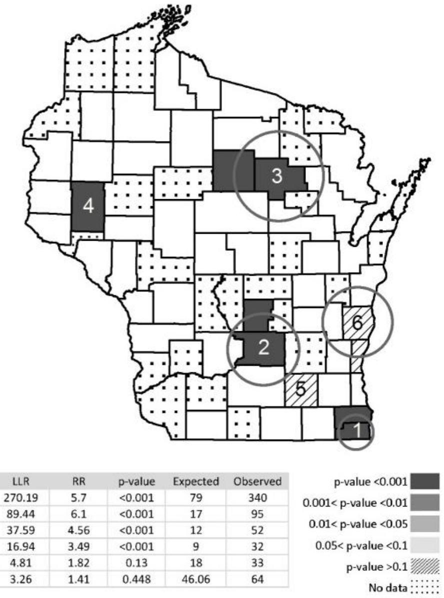

Cluster	LLR	RR	p-value	Expected	Observed
1	270.19	5.7	<0.001	79	340
2	89.44	6.1	<0.001	17	95
3	37.59	4.56	<0.001	12	52
4	16.94	3.49	<0.001	9	32
5	4.81	1.82	0.13	18	33
6	3.26	1.41	0.448	46.06	64
