# Supplementary material for: Gray Wolf Exposure to Emerging Vector-Borne Diseases in Wisconsin with Comparison to Domestic Dogs and Humans
Source: PLoS One. 2016 Nov 29;11(11):e0165836. doi: 10.1371/journal.pone.0165836 (PMC5127498; doi:10.1371/journal.pone.0165836)

S2 Fig. Gray wolf and dog percent positive test results (number positive/number tested) for antibodies to *Borrelia burgdorferi*, *Anaplasma phagocytophilum* and *Echrlichia canis* and antigen of *Dirofilaria immitis* in Wisconsin between 2001 and 2007. Wolf exposure to each pathogen were siginificantly greater than dog exposure at α=0.05 level. Sample size for wolves=178-178 and dogs=51512-109745 depending on the pathogen.

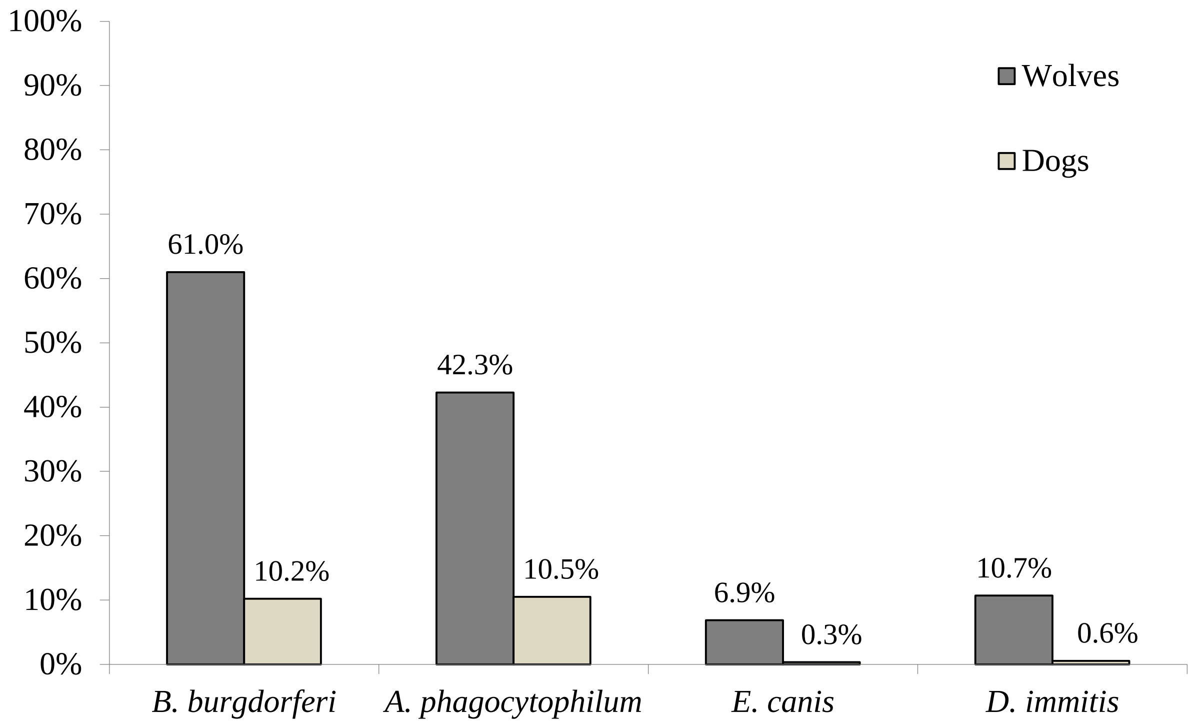

Supplement: S2 Fig — Wolf exposure to each pathogen were siginificantly greater than dog exposure at α = 0.05 level. Sample size for wolves = 178–178 and dogs = 51512–109745 depending on the pathogen. (PDF) [file pone.0165836.s003.pdf]
