# Supplementary material for: Gray Wolf Exposure to Emerging Vector-Borne Diseases in Wisconsin with Comparison to Domestic Dogs and Humans
Source: PLoS One. 2016 Nov 29;11(11):e0165836. doi: 10.1371/journal.pone.0165836 (PMC5127498; doi:10.1371/journal.pone.0165836)
Supplement: S1 Text — (PDF) [file pone.0165836.s004.pdf]

S1 Text

Because the SNAP 4Dx Test was designed for domestic dogs, we conducted a validation study for gray wolves to determine if the SNAP 4Dx Test can be used to assess disease exposure/infection in gray wolves (*Canis lupus*). We identified 36 wolf sera samples previously tested for at least one of the four pathogens in this study. An additional 11 wolves were heartworm positive at necropsy. We compared wolf serum SNAP 4Dx Test results to previous results obtained with different diagnostic tests for at least one of the four diseases. *B. burgdorferi* and *A. phagocytophilum* were previously analyzed by Marshfield Medical Center Laboratory in Wisconsin utilizing an indirect immunofluorescent antibody (IFA) assay. For *D. immitis*, samples were analyzed by Marshfield Medical Center Laboratory using a commercial ELISA (DiroCHEK), or by the Wisconsin Veterinary Diagnostic Laboratory (WVDL) – Madison or an unknown laboratory (records lost) using an ELISA for detection of *D. immitis* uterine antigen. We also used wolves diagnosed with heartworm at necropsy (based on observation and identification of worms). We compared the SNAP 4Dx Test results to the previous laboratory results and to necropsy results for *D. immitis*. For each pathogen we calculated the percentage of concordance (both positive and negative results) between the SNAP 4Dx Test and previous laboratory or necropsy results. We used a binomial test to evaluate agreement where a significant P-value indicates disagreement. If the binomial test was not significant we assessed the level of agreement utilizing a Kappa statistic (<0.2 indicates slight, 0.2 to 0.4 fair, 0.4 to 0.6 moderate, 0.6 to 0.8 substantial, >0.8 almost perfect agreement) [4].

Unfortunately, none of the sera samples was previously tested for antibodies against *E. canis*. We found *B. burgdorferi* had the highest concordance (94.1% n=17) with almost perfect agreement between both diagnostic tests (P=1; Kappa=0.77; SE of Kappa= 0.22) (Positive-Positive= 14, Negative-Negative= 2, Negative-Positive=1, Positive-Negative=0; SNAP 4Dx Test results second). There was weak agreement for anaplasmosis (P=0.031, Kappa=0.16; SE for Kappa= 0.15) (Positive-Positive= 10, Negative-Negative= 1, Negative-Positive=0, Positive-Negative=6; SNAP 4Dx Test results second) with concordance of 64.7% for 17 samples. Finally, for heartworm infection there was 92% concordance (n=24, P=0.75); however, there were few positive heartworm serology tests and agreement was poor (Kappa=-0.06; SE for Kappa= 0.04) (Positive-Positive= 0, Negative-Negative= 22, Negative-Positive=2, Positive-Negative=1; SNAP 4Dx Test results second). Of the 11 heartworm positive wolves at necropsy, only 2 (18.2%) tested positive with the SNAP 4Dx Test.

The near perfect agreement with prior laboratory results for Lyme disease indicates that the SNAP 4Dx Test performs well for detecting antibodies against *B. burgdorferi* in wolf blood/serum. However, agreement was lower for anaplasmosis (64.7% concordance) suggesting the SNAP 4Dx Test has lower sensitivity in wolves than previously described for dogs [3]. Concurrence for heartworm results may be misleading due to the small number of positive samples for both tests. In addition, < 20% of the wolves with heartworms observed during necropsy were positive on the SNAP 4Dx Test, indicating the test has lower sensitivity than previously determined in dogs with low worm burdens [1]. These results suggest that the SNAP 4Dx Test might underestimate true prevalence of exposure to *A. phagocytophilum* and infection with *D. immitis* in wolves. Alternatively, sensitivity may be lost during longer storage times that

elapsed between SNAP 4Dx Testing of wolf samples and previously conducted evaluations [2] and we believe these topics deserve further research consideration.

References

1. Atkins CE. Comparison of results of three commercial heartworm antigen test kits in dogs with low heartworm burdens. *J Am Vet Med Assoc* 2003;222: 1221-1223

2. Curry PS, Elkin BT, Campbell M, Nielsen K, Hutchins W, Ribble C, Kutz SJ. Filter-paper blood samples for ELISA detection of *Brucella* antibodies in caribou. *J Wildl Dis* 2011;47: 12-20.

3. Chandrashekar R, Mainville CA, Beall MJ, O'Connor T, Eberts MD, Alleman AR, Gaunt SD, Breitschwerdt EB. Performance of a commercially available in-clinic ELISA for the detection of antibodies against *Anaplasma phagocytophilum*, *Ehrlichia canis*, and *Borrelia burgdorferi* and *Dirofilaria immitis* antigen in dogs. *Am J Vet Med* 2010;71: 1443-1450.

4. Dohoo I, Martin W, Stryhn H. *Veterinary epidemiologic research*, AVC Inc., Charlottetown, Prince Edward island, Canada; 2003. 706 pp.
